# Three new species and the molecular phylogeny of *Antipathozoanthus* from the Indo-Pacific Ocean (Anthozoa, Hexacorallia, Zoantharia)

**DOI:** 10.3897/zookeys.725.21006

**Published:** 2017-12-29

**Authors:** Hiroki Kise, Takuma Fujii, Giovanni Diego Masucci, Piera Biondi, James Davis Reimer

**Affiliations:** 1 Molecular Invertebrate Systematics and Ecology Laboratory, Graduate School of Engineering and Science, University of the Ryukyus, 1 Senbaru, Nishihara, Okinawa 903-0213, Japan; 2 Palau International Coral Reef Center, 1-M-Dock Road, Koror, Palau 96940; 3 Research Center for Island Studies Amami Station, Kagoshima University, Naze-Yanagimachi 2-1, Amami, Kagoshima 894-0032, Japan; 4 Tropical Biosphere Research Center, University of the Ryukyus, 1 Senbaru, Nishihara, Okinawa 903-0213, Japan

**Keywords:** antipatharian, cave-dwelling, diversity, evolution, new species, substrate specificity

## Abstract

In this study, three new species of macrocnemic zoantharians (Hexacorallia, Zoantharia) are described from localities in the Indo-Pacific Ocean including the Red Sea, the Maldives, Palau, and southern Japan: *Antipathozoanthus obscurus* **sp. n.**, *A. remengesaui* **sp. n.**, and *A. cavernus* **sp. n.** Although the genus *Antipathozoanthus* is currently restricted to species living on antipatharians, *A. obscurus* **sp. n.** is not associated with any living substrate and instead is found on coral reef carbonate substrate within narrow caves or cracks. The two new species that have association with antipatharians, *A. remengesaui* **sp. n.** and *A. cavernus* **sp. n.**, can be distinguished by their relative coenenchyme development and the antipatharian species that each uses as substrate. Additionally, all new species described in this study have unique nuclear internal transcribed spacer region of ribosomal DNA (ITS-rDNA) sequences. Our results indicate that more phylogenetic studies focusing on increasing the numbers of species examined within each of the genera of Parazoanthidae are required in order to better understand the evolutionary history of substrate specificity within the family Parazoanthidae.

## Introduction

Zoantharia Rafinesque, 1815 is the third most speciose order within the subclass Hexacorallia Haeckel, 1896. Zoantharians can be found in a wide variety of marine environments from intertidal zones to deep-sea cold seeps (e.g., Reimer et al. 2007b), and are characterized by having two rows of tentacles and the unique bilateral arrangements of the mesenteries, with most species forming clonal colonies without hard structures such as skeletons of the order Scleractinia. Zoantharia is currently divided into two suborders; Brachycnemina Haddon & Shackleton, 1891, and Macrocnemina Haddon & Shackleton, 1891, based on differences in the fifth pair of mesenteries from the dorsal directive. Zoantharians within suborder Macrocnemina are distributed worldwide, and are usually found in associations with other invertebrates. Within Macrocnemina, the largest family is Parazoanthidae Delage & Hérouard, 1901, which currently contains 13 genera (Low et al. 2016). Most species of these genera live in association with other marine invertebrates, including antipatharians (Ocaña and Brito 2003; Sinniger et al. 2010), octocorals (Reimer et al. 2008; Bo et al. 2012; Sinniger et al. 2013), and sponges (Haddon and Shackleton 1891; Swain and Wulff 2007; Montenegro et al. 2015, 2016). Historically, establishing the taxonomic framework of Parazoanthidae was challenging due to relatively few diagnostic morphological characteristics (Sinniger et al. 2005; Montenegro et al. 2015), and the family was shown to be paraphyletic in initial molecular studies (Sinniger et al. 2005). Recently, however, studies based on molecular phylogeny combined with ecological data have greatly revised the taxonomy within the family Parazoanthidae (Sinniger et al. 2005, 2013; Sinniger and Häussermann 2009; Montenegro et al. 2015, 2016). As a consequence of these studies, nine genera within Parazoanthidae have been described since 2008 and another genus, *Bergia* Duchassaing & Michelotti, 1860, has been resurrected. Key to this new taxonomic framework is the idea initially proposed by Sinniger et al. (2005, 2010) that different parazoanthid genera share long evolutionary histories with the associated marine invertebrates they use as substrates.

One of these recently erected genera is *Antipathozoanthus* Sinniger, Reimer & Pawlowski, 2010. As the generic name indicates, species in this genus utilize antipatharians (Hexacorallia, Antipatharia) as their obligate substrate. The genus currently includes two valid species; *A. macaronesicus* (Ocaña & Brito, 2003) from the eastern Atlantic and *A. hickmani* Reimer & Fujii, 2010 from the Galapagos Islands. Additionally, several potentially undescribed species have been reported from the Red Sea (Reimer et al. 2014b), the South China Sea (Reimer et al. 2017), and Japan (Sinniger et al. 2010; Reimer et al. 2013, 2014a). However, the species diversity of *Antipathozoanthus* spp. in the Indo-Pacific Ocean remains generally unknown. In this study, three new *Antipathozoanthus* species are formally described based on specimens collected from a number of regions in the Indo-Pacific Ocean, and the genus is redescribed based on these findings.

## Materials and methods

**Figure 1.**
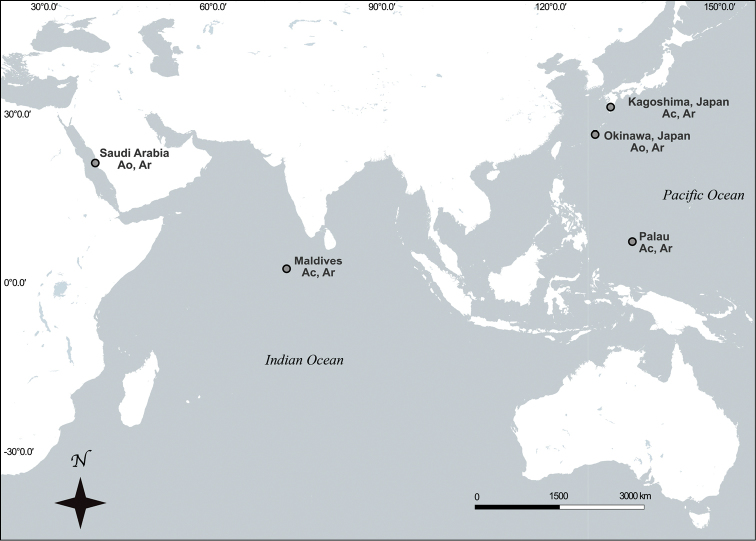
Sampling location in the Indian Ocean and Pacific Ocean of specimens used in this study. Location of specimens collected in this study represented by closed symbols. Species abbreviations after locations: (Ao) *Antipathozoanthus obscurus* sp. n.; (Ar) *A. remengesaui* sp. n.; (Ac) *A. cavernus* sp. n.

**Specimen collection.** *Antipathozoanthus* specimens were collected between 2009 to 2016 from three localities in the Red Sea, three localities in the Maldives, five localities in Japan, and two localities in Palau (Fig. 1), with one comparative specimen of *A. macaronesicus* collected from Pico Island, Azores, Portugal. All specimens were collected by SCUBA. Specimen images were taken *in situ* for gross external morphological analyses. Collected specimens were preserved in 99.5% ethanol (Table 1).

**Table 1.** List of examined specimens, and GenBank Accession Numbers.

Specimen ID	Genus	Species	Locality	Coordinates		Collecter	Sampling date	Depth (m)	Accession number (COI)	Accession number (16S–rDNA)	Accession number (ITS–rDNA)
				Latitude / Longitude							
AZCN	*Antipathozoanthus*	*macaronesicus*	Pico Island, Azores, Portugal	N38°28"3.8", W28°24′0″		P Wirtz	13-May-16	43	MG384664	MG384684	MG384696
BISE1	*Antipathozoanthus*	*obscurus*	Bise, Motobu, Okinawa, Japan	N26°42'34.4", E127°52'49.2"		JD Reimer, I Kawamura	14-Aug-14	5	MG384644	MG384685	MG384691
BISE3	*Antipathozoanthus*	*obscurus*	Bise, Motobu, Okinawa, Japan	N26°42'34.4", E127°52'49.2"		JD Reimer, I Kawamura	14-Aug-14	5	–	–	MG384693
MAL46	*Antipathozoanthus*	*remengesaui*	Coral Garden, Maldives	N3°05'24.3", E72°58'04.5"		JD Reimer	06-May-14	24	MG384658	MG384679	–
MAL82	*Antipathozoanthus*	*remengesaui*	Wall Street, Maldives	N3°07'14.2", E72°58'46.5"		JD Reimer	07-May-14	9	MG384657	–	–
MAL83	*Antipathozoanthus*	*remengesaui*	Wall Street, Maldives	N3°07'14.2", E72°58'46.5"		JD Reimer	07-May-14	9	MG384656	–	–
MAL84	*Antipathozoanthus*	*remengesaui*	Wall Street, Maldives	N3°07'14.2", E72°58'46.5"		JD Reimer	07-May-14	9	MG384655	–	MG384702
MAL85	*Antipathozoanthus*	*remengesaui*	Wall Street, Maldives	N3°07'14.2", E72°58'46.5"		JD Reimer	07-May-14	9	MG384654	MG384678	MG384701
MAL145	*Antipathozoanthus*	*remengesaui*	Wall Street, Maldives	N3°07'14.2", E72°58'46.5"		JD Reimer	10-May-14	12	MG384653	MG384677	–
MAL147	*Antipathozoanthus*	*remengesaui*	Wall Street, Maldives	N3°07'14.2", E72°58'46.5"		JD Reimer	10-May-14	10	MG384652	–	–
MAL2592601	*Antipathozoanthus*	*cavernus*	Capital Reef, Maldives	N3°02'55.8", E72°53'21.2"		M Oliverio	16-May-14	19	MG384651	MG384676	MG384697
MAL2592602	*Antipathozoanthus*	*remengesaui*	Capital Reef, Maldives	N3°02'55.8", E72°53'21.2"		M Oliverio	16-May-14	19	–	MG384675	–
MAL261	*Antipathozoanthus*	*remengesaui*	Wall Street, Maldives	N3°07'14.2", E72°58'46.5"		JD Reimer	17-May-14	9	MG384650	MG384674	–
KINKO1	*Antipathozoanthus*	*cavernus*	Sakurajima, Kagoshima, Japan	N31°35'23.5", E130°35.27.8"		JD Reimer	20-Sep-15	21	MG384660	MG384681	MG384699
KINKO2	*Antipathozoanthus*	*remengesaui*	Sakurajima, Kagoshima, Japan	N31°35'23.5", E130°35'27.8"		JD Reimer	20-Sep-15	21	MG384659	MG384680	–
PALAU2	*Antipathozoanthus*	*remengesaui*	Blue Hole, Palau	N7°8'29.4", E134°13'23.3"		JD Reimer	15-Sep-14	23	MG384649	MG384673	MG384703
PALAU3	*Antipathozoanthus*	*remengesaui*	Siaes Tunnel. Palau	N7°18'54.8", E134°13'13.3"		JD Reimer	15-Sep-14	37	MG384648	–	–
PALAU4	*Antipathozoanthus*	*remengesaui*	Blue Hole, Palau	N7°8'29.4", E134°13'23.3"		JD Reimer	12-Sep-14	28	MG384647	MG384672	–
PALAU5	*Antipathozoanthus*	*cavernus*	Siaes Tunnel. Palau	N7°18'54.8", E134°13'13.3"		JD Reimer	15-Sep-14	39	–	–	MG384698
HK70	*Antipathozoanthus*	*remengesaui*	Siaes Tunnel. Palau	N7°18'54.8", E134°13'13.3"		H Kise	12-Sep-14	NA	MG384663	MG384683	–
HK90	*Antipathozoanthus*	*remengesaui*	Blue Hole, Palau	N7°8'29.4", E134°13'23.3"		H Kise	15-Sep-14	22	MG384662	–	–
TF54	*Antipathozoanthus*	*obscurus*	Cape Zanpa, Yomitan, Okinawa, Japan	N26°26'26.5", E127°42'43.7"		T Fujii	06-Apr-09	3	MG384641	–	MG384689
TF78	*Antipathozoanthus*	*obscurus*	Cape Manza, Onna, Okinawa, Japan	N26°30'18.3", E127°51'02.3"		T Fujii	02-Oct-09	5	MG384640	MG384668	MG384687
TF102	*Antipathozoanthus*	*remengesaui*	Sakurajima, Kagoshima, Japan	N31°35'23.5", E130°35'27.8"		T Fujii	26-Jul-11	20	MG384646	–	MG384704
TF103	*Antipathozoanthus*	*remengesaui*	Sakurajima, Kagoshima, Japan	N31°35'23.5", E130°35'27.8"		T Fujii	26-Jul-11	40	MG384645	–	MG384705
TF148	*Antipathozoanthus*	*obscurus*	Cape Manza, Yomitan, Okinawa, Japan	N26°30'18.3", E127°51'02.3"		T Fujii	22-Oct-12	10	MG384642	MG384669	MG384688
TF173	*Antipathozoanthus*	*remengesaui*	Onna, Okinawa, Japan	N26°26'20.9", E127°47'7.2"		T Fujii	27-Jun-14	15	–	–	–
JDR190	*Antipathozoanthus*	*obscurus*	Al Wajh Shaybarah, Saudi Arabia	N25°21', E36°54'		JD Reimer	03-Oct-13	3	–	MG384667	MG384692
JDR191	*Antipathozoanthus*	*obscurus*	Al Wajh Shaybarah, Saudi Arabia	N25°21', E36°54'		JD Reimer	03-Oct-13	3	–	MG384666	MG384694
JDR192	*Antipathozoanthus*	*obscurus*	Al Wajh Shaybarah, Saudi Arabia	N25°21', E36°54'		JD Reimer	03-Oct-13	3	MG384643	MG384665	MG384695
JDR209	*Antipathozoanthus*	*remengesaui*	Yanbu , Saudi Arabia	N24°26', E37°14'		JD Reimer	04-Oct-13	11	–	–	MG384700
JDR211	*Antipathozoanthus*	*remengesaui*	Yanbu , Saudi Arabia	N24°26', E37°14'		JD Reimer	04-Oct-13	12	–	MG384682	–
JDR214	*Antipathozoanthus*	*remengesaui*	Yanbu , Saudi Arabia	N24°26', E37°14'		JD Reimer	04-Oct-13	12	MG384661	–	–
JDR279	*Antipathozoanthus*	*obscurus*	Shib Nazar, Saudi Arabia	N22°19', E38°51'		JD Reimer	10-Oct-13	4	–	MG384671	MG384690
KU1	*Antipathozoanthus*	*obscurus*	Ara, Kumejima Island, Okinawa, Japan	N26°19'15.0", E126°45'21.3"		T Fujii	20-Nov-09	15	MG384639	MG384670	MG384686

**Molecular analyses.** *Antipathozoanthus* DNA was extracted using the guanidine protocol following Sinniger et al. (2010). PCR was performed for three genetic markers: mitochondrial cytochrome oxidase subunit I (COI), mitochondrial 16S ribosomal DNA (16S-rDNA), and the nuclear internal transcribed spacer region of ribosomal DNA (ITS-rDNA) using a HotStarTaq Master Mix Kit (Qiagen, Tokyo, Japan). COI was amplified with the following primers: COIZoanF (5’-TGA TAA GGT TAG AAC TTT CTG CCC CGG AAC-3’) (Reimer et al. 2007b) and COIantr (5’-GCC CAC ACA ATA AAG CCC AA TAY YCC AAT-3’) (Sinniger et al. 2010). 16S-rDNA was amplified with the following primers: 16SarmL (5’-GGC CTC GAC TGT TTA CCA AA-3’) (Fujii and Reimer 2011) and 16SbmoH (5’-CGA ACA GCC AAC CCT TGG-3’) (Sinniger et al. 2005). The ITS-rDNA was amplified with the following primer pairs: either ITSf (5’-CTA GTA AGC GCG AGT CAT CAG C-3’) and ITSr (5’-GGT AGC CTT GCC TGA TCT GA-3’) (both Swain 2009) or Zoan-f (5’-CTT GAT CAT TTA GAG GGA GT-3’) and Zoan-r (5’-CGG AGA TTT CAA ATT TGA GCT-3’) (both Reimer et al. 2007a). The markers were amplified following the thermal cycle conditions: 5 min at 95 °C followed by 35 cycles of: 30 s at 94 °C, 1 min at 40 °C, and 1 min 30 s at 72 °C, and followed by a 7 min extension at 72 °C for COI; 5 min at 95 °C and then 35 cycles of: 1 min at 95 °C, 1 min at 52 °C, and 2 min at 72 °C, followed by a 7 min extension at 72 °C for 16S-rDNA; and 5 min at 95 °C then 35 cycles of: 1 min at 94 °C, 1 min at 50 °C, and 2 min at 72 °C, followed by a 10 min extension at 72 °C for ITS-rDNA. Amplified PCR products were checked by 1.5 % agarose gel and positive PCR products were sequenced in both directions by Fasmac (Kanagawa, Japan) after clean up using shrimp alkaline phosphatase (SAP) and Exonuclease I (Takara Bio Inc., Shiga, Japan).

**Molecular phylogenetic analyses**. Newly obtained sequences were inspected by eye and manually edited using Geneious v8.1 (Kearse et al. 2012, http://www.geneious.com) and deposited in GenBank (accession numbers MG384639–MG384705; Table 1). Nucleotide sequences of COI, 16S-rDNA and ITS-rDNA from specimens were aligned with previous study sequences from various parazoanthid genera (*Antipathozoanthus*, *Bergia*, *Bullagummizoanthus*, *Corallizoanthus*, *Hurlizoanthus*, *Kauluzoanthus*, *Kulamanamana*, *Mesozoanthus*, *Parazoanthus*, *Umimayanthus*, *Zibrowius*) using the Muscle algorithm (Geneious plug-in; Edgar 2004) (Suppl. material 1). Sequences of the genus *Epizoanthus* were selected as the outgroup for all three markers’ alignments. The *A. hickmani* sequence from Reimer and Fujii (2010; EU333790) was not included in the COI phylogenetic tree in this study due to its short length (280 bp). The 16S-rDNA and ITS-rDNA indels were aligned following previous studies (Sinniger et al. 2010; Montenegro et al. 2016). Three alignment datasets were generated; 430 sites of 48 sequences for COI; 589 sites of 57 sequences for 16S-rDNA and 938 sites of 48 sequences for ITS-rDNA. The alignment data are available as electronic supplementary material (Suppl. material 1–4).

The generated alignments of each marker were used to construct a concatenated alignment. All missing data, including gaps, were replaced with “N". All specimens of *Antipathozoanthus* included in the concatenated alignment included at least ITS-rDNA sequences. The concatenated alignment consisted of 1957 positions and 54 sequences. Phylogenetic analyses of the concatenated alignment were performed using maximum likelihood (ML) and Bayesian inference (BI), with gene partitions set for ML in RAxML v8 (Stamatakis 2014), and gene partitions for BI as indicated by jModelTest version 0.0.1 (Posada 2008) per each marker in MrBayes v3.2.2 (Huelsenbeck and Ronquist 2001) as shown below. Phylogeny reconstructions were performed for each marker using neighbor joining (NJ), ML and BI.

The NJ phylogeny reconstruction was performed using Geneious v8.1 (Kearse et al. 2012, http://www.geneious.com) with the Hasegawa-Kishino-Yano genetic distance model (HKY) (Hasegawa et al. 1985) and 1000 replicates of bootstrapping. The best-fitting models for ML phylogeny reconstruction were performed by jModelTest under Akaike Information Criterion (AIC). The following models were suggested by jModelTest: TrN+I for the COI dataset; K80+G for the 16S-rDNA dataset; HKY+I+G for ITS-rDNA dataset. ML phylogenetic trees were constructed with PhyML (Guindon and Gascuel 2003) for each marker independently. PhyML was performed using an input tree generated by BIONJ with the models suggested by jModelTest, with 8 gamma-categories of substitution rates. Bootstrap replicates (1000) were conducted using the same parameters. The best fitting models for BI phylogeny reconstruction was performed by jModelTest under Bayesian Information Criterion (BIC). The following models were suggested by jModelTest: K80+G for the COI dataset; K80+G for the 16S-rDNA dataset; and HKY+I+G for the ITS-rDNA dataset. BI phylogenetic trees were constructed with the program MrBayes as a plug-in in Geneious with the models suggested by jModelTest. One cold and three heated Markov chain Monte Carlo (MCMC) chains with default temperature were run for 20,000,000 generations, subsampling frequency of 1000 and a burn in length of 3,000,000 (15%) for all alignments. Average Standard Deviation of Split Frequency (ASDOSF) values were <0.01 for all three Bayesian datasets.

**Morphological analyses**. Numbers of tentacles, polyp coloration, oral disk coloration, relative tentacle lengths, and polyp dimensions (oral disk diameter/polyp height) were examined using *in situ* images. Additionally, the relative development of the coenenchyme was examined using a dissecting microscope. Coenenchyme development was classified as 1) “highly developed coenenchyme" when polyps covered the antipatharian substrate completely, or 2) “poorly developed coenenchyme" when polyps did not completely cover the antipatharian substrate and the antipatharians were clearly visible. For internal morphological analyses, we observed mesentery arrangement and numbers, and location and shape of marginal muscle. Histological sections of 8 µm thickness were made and stained with hematoxylin and eosin after decalcification with Bouin’s fluid for 24h.

**Cnidae analyses**. Cnidae analyses were conducted using undischarged cnidocysts from tentacles, column, actinopharynx, and mesenteries filaments of holotype polyps (n = 6) for all new species under a Nikon Eclipse80i stereomicroscope (Nikon, Tokyo). Cnidae sizes were measured using ImageJ v1.45s (Rasband 2012). Although cnidae classification basically followed England (1991) and Ryland and Lancaster (2004), basitrichs and microbasic mastigophores were considered as the same type of nematocyst based on studies by Schmidt (1974), Hidaka et al. (1987), and Hidaka (1992), and therefore these two types were pooled together in this study.

### Abbreviations used

**NSMT** National Science Museum, Tsukuba, Ibaraki, Japan

**RMNH** Naturalis Biodiversity Center, Leiden, Netherlands

**RUMF** Ryukyu University Museum, Fujukan, University of the Ryukyus, Nishihara, Okinawa, Japan

**MISE** Molecular Invertebrate Systematics and Ecology Laboratory, University of the Ryukyus, Nishihara, Okinawa, Japan

## Results

### Systematics

#### Phylum Cnidaria Hatschek, 1888

##### Class Anthozoa Ehrenberg, 1831

###### Subclass Hexacorallia Haeckel, 1896

####### Order Zoantharia Rafinesque, 1815

######## Suborder Macrocnemina Haddon & Shackleton, 1891

######### Family Parazoanthidae Delage & Hérouard, 1901

########## Antipathozoanthus

Taxon classificationAnimaliaZoanthariaParazoanthidae

Sinniger, Reimer & Pawlowski, 2010

########### Type species.

*Antipathozoanthus macaronesicus* (Ocaña & Brito, 2003)

########### Diagnosis.

Macrocinemic zoantharians with cteniform endodermal muscle or endo-meso transitional sphincter muscle (Swain et al. 2015). Substrate consists of either antipatharians or coral carbonate (reef). Genetic distance of mitochondrial COI sequences and insertion/deletion patterns in 16S-rDNA sequences are significantly different from those in other parazoanthid genera (Sinniger et al. 2005, 2010).

########### Remarks.

Four of five formally described species grow mainly on antipatharians, but this character is not exclusive to all species in the genus as *A. obscurus* sp. n. is not associated with any host organism. Results of the current study showed that *A. obscurus* sp. n. is clearly placed within this genus according to COI and 16S-rDNA sequence analyses. Thus, these non-associated species/specimens are within the genus based on their phylogenetic position but do not fit the original definition of the genus by Sinniger et al. (2010).

########## Antipathozoanthus obscurus sp. n.

Taxon classificationAnimaliaZoanthariaParazoanthidae

http://zoobank.org/2CE5BEAD-1772-4CB6-A7DA-EEA2FB480F87

Fig. 2a, b

Antipathozoanthus sp. 3 *sensu*Reimer and Fujii 2017, 394, fig. 14.4e.

########### Material examined.

*Holotype*: NSMT-Co1602 (MISE-BISE1), collected from the wall of a shallow cave in a coral reef. Preserved polyps are approximately 3.0–4.5 mm in diameter, and approximately 3.0–8.0 mm in height from the coenenchyme. Approximately 15–20 polyps connected by a stolon form a mesh network, with additional solitary polyps close by (n = 6). Polyps and coenenchyme are heavily encrusted by various fine sand particles. External color light orange when alive, light beige when fixed. Collected from Cape Bise, Motobu, Okinawa-jima Island, Japan (26°42'34.4"N, 127°52'49.2"E) at a depth of 5 m by James Davis Reimer (JDR), 14 August 2014.

*Paratypes*: RUMF-ZG-4390 (MISE-JDR190), collected from Al Wajh Shaybarah, Saudi Arabia, (25°21'N, 36°54'E) at a depth of 3 m by JDR, 3 October 2013; RUMF-ZG-4391 (MISE-JDR191), collected from Al Wajh Shaybarah, Saudi Arabia, (25°21'N, 36°54'E) at a depth of 3 m by JDR, 3 October 2013; RUMF-ZG-4392 (MISE-JDR192), collected from Al Wajh Shaybarah, Saudi Arabia, (25°21'N, 36°54'E) at a depth of 3 m by JDR, 3 October 2013; RUMF-ZG-4393 (MISE-JDR279), collected from Shib Nazar, Saudi Arabia, (22°19'N, 38°51'E) at a depth of 3 m by JDR, 3 October 2013; RUMF-ZG-4394 (MISE-KU1), collected from Kume-jima Island, Okinawa, Japan (26°19'15.0"N, 126°45'21.3"E) at a depth of 15 m by Takuma Fujii (TF), 20 November 2009; RUMF-ZG-4395 (MISE-TF54), collected from Cape Zanpa, Yomitan, Okinawa-jima Island, Japan (26°26'26.5"N, 127°42'43.7"E) at a depth of 3 m by TF, 6 April 2009, divided into two pieces, one portion fixed in 99.5% ethanol, and other in 5–10% saltwater formalin; RUMF-ZG-4396 (MISE-TF78), collected from Cape Manza, Onna, Okinawa-jima Island, Japan (26°30'18.3"N, 127°51'02.3"E) at a depth of 5 m by TF, 2 October 2009, divided into two pieces, one portion fixed in 99.5% ethanol, and other in 5–10% saltwater formalin; RMNH.Coel.42320 (MISE-TF148), collected from Cape Manza, Onna, Okinawa-jima Island, Japan (26°30'18.3"N, 127°51'02.3"E) at a depth of 10 m by TF, 22 October 2012.

*Other materials examined*: MISE-BISE3, collected from Cape Bise, Motobu, Okinawa-jima Island, Japan (26°42'34.4"N, 127°52'49.2"E) at a depth of 5 m by JDR, 14 August 2014.

########### Diagnosis.

**Figure 2.**
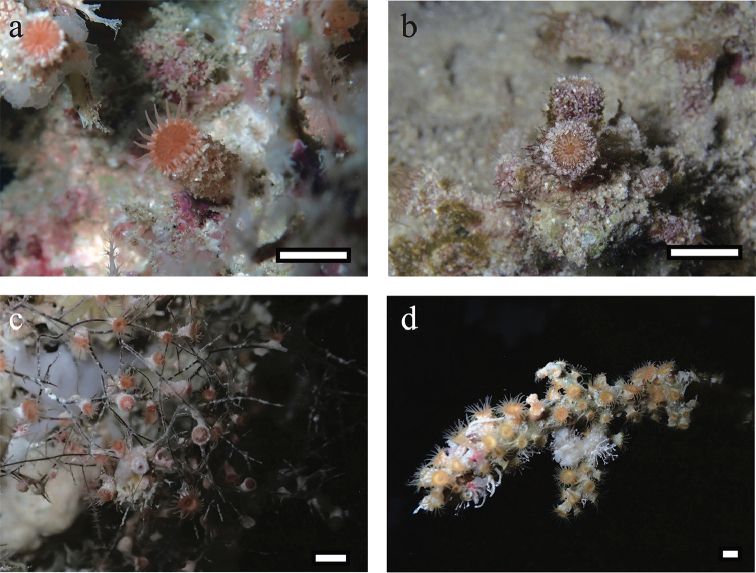
Polyp images of *Antipathozoanthus obscurus* sp. n., *A. remengesaui* sp. n. and *A. cavernus* sp. n. *in situ*. **a** *A. obscurus* sp. n. . NSMT-Co1602 (MISE-BISE1), Collected from Cape Bise, Motobu, Okinawa-jima Island, Japan (26°42'34.4"N, 127°52'49.2"E) at a depth of 5 m by JDR, 14 August 2014. **b** *A. obscurus* sp. n., closed polyp with heavy encrustion by various fine sand particles. MISE-TF54, collected from Cape Zanpa, Yomitan, Okinawa-jima Island, Japan (26°26'26.5"N, 127°42'43.7"E) at a depth of 3 m by TF, 6 April 2009. **c** *A. remengesaui* sp. n., colony connected by poorly developed coenenchyme with white polyps on *Antipathes* sp. NSMT-Co1603 (MISE-PALAU2) collected from Blue Hole, Palau (7°8'29.4"N, 134°13'23.3"E) at a depth of 23 m by JDR, 15 September 2014 **d** *A. cavernus* sp. n., polyp connected by highly developed coenenchyme with orange ring around oral disk. RMNH.Coel.42322 (MISE-PALAU5) collected from Siaes Tunnel, Palau (7°18'54.8"N, 134°13'13.3"E) at a depth of 39 m by JDR, 1 September 2014.

*External morphology*: Open oral disks are approximately 5–10 mm in diameter, and polyps approximately 5–10 mm in height when open (Fig. 2). Polyps of a single colony are usually connected by a stolon forming a mesh-like network. *Antipathozoanthus obscurus* sp. n. has approximately 26–32 bright brown and/or orange tentacles that are as long as or longer than oral disk diameter. Polyps and coenenchyme have a heavily encrusted ectoderm including numerous various sand particles (usually 1 to 8 mm in size). Capitular ridges (= number of complete mesenteries) are slightly visible on tops (= capitulum) of closed polyps.

*Internal morphology*: Azooxanthellate. Fine sand particles and silica heavily encrusted into ectoderm and mesoglea. We could not obtain cross-sections or images to observe internal morphology such as mesenterial arrangement, marginal muscle or siphonoglyph due to heavy sand and silica encrustation.

**Figure 3.**
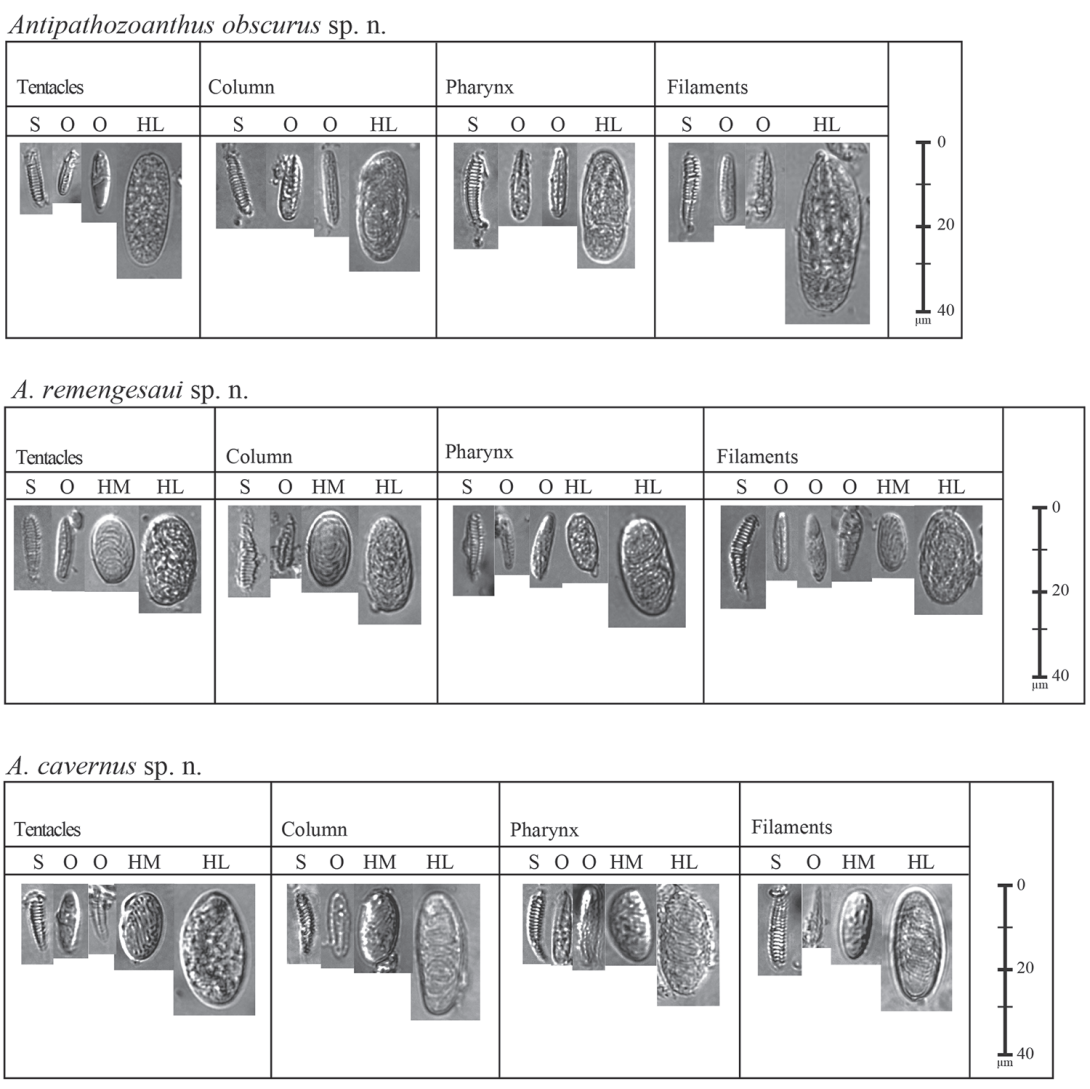
Cnidae in the tentacles, column, pharynx, and filament of *Antipathozoanthus obscurus* sp. n., *A. remengesaui* sp. n. and *A. cavernus* sp. n., respectively. Abbreviations: (HL) holotrich large, (HM) holotrich medium, (O) bastrichs or mastigophores, (S) spriocysts.

*Cnidae*: Holotrichs (large), basitrichs and microbasic p-mastigophores (usually difficult to distinguish), spirocysts (Fig. 3; Table 2).

**Table 2.** Cnidae types and sizes observed in three new Antipathozoanthus species. Frequency: relative abundance of cnidae type in decreasing order; numerous, common, occasional, rare (n = number of cnidae).

		*Antipathozoanthus obscurus* sp. n.				*A. remengesaui* sp. n.				*A. cavernus* sp. n.			
		Length (min-max, average)	Width (min-max, average)	n	Frequency	Length (min-max, average)	Width (min-max, average)	n	Frequency	Length (min-max, average)	Width (min-max, average)	n	Frequency
Tentacles	Spirocysts	11–20, 16.3	2–5, 3.1	48	Occasional	11–25, 18.0	2–7, 3.4	98	Numerous	14–25, 18.0	2–5, 2.9	137	Numerous
	Holotrichs (L)	21–33, 28.1	10–15, 11.9	39	Occasional	20–29, 21.8	7–14, 10.1	27	Occasional	20–31, 22.7	9–17, 11.4	28	Occasional
	Holotrichs (M)	–	–	–	–	10–19, 17.0	5–17, 9.9	105	Numerous	14–19, 17.9	7–14, 10.0	95	Numerous
	Bastrichs and Mastigophores	–	–	–	–	10–23, 16.3	2–6, 4.2	31	Occasional	15–19, 16.7	4–6, 4.7	7	Rare
													
Column	Spirocysts	12–22, 18.2	2–5, 3.1	57	Numerous	17–25, 19.7	2–4, 3.1	20	Occasional	11–28, 17.5	2–16, 5.6	44	Numerous
	Holotrichs (L)	22–34, 28.2	9–15, 11.8	78	Numerous	20–29, 25.1	9–17, 12.5	40	Occasional	20–32, 25.6	9–16, 11.2	24	Occasional
	Holotrichs (M)	–	–	–		11–19, 17.0	8–12, 9.9	21	Occasional	12–19, 16.5	5–13, 8.8	40	Occasional
	Bastrichs and Mastigophores	12–25, 18.2	2–5, 3.6	29	Occasional	14–18, 15.9	4–8, 5.9	16	Common	2	5	1	Rare
													
Pharynx	Spirocysts	13–25. 16.5	2–5, 3.0	69	Numerous	11–24, 17.7	2–6, 3.4	65	Numerous	13–23, 18.2	2–5, 3.2	101	Numerous
	Holotrichs (L)	20–34, 28.5	8–15, 11.5	76	Numerous	20–31, 23.4	7–18, 11.7	35	Occasional	20–31, 22.7	9–15, 12.0	33	Occasional
	Holotrichs (M)	–	–	–		10–19, 16.8	6–13, 9.5	76	Numerous	13–19, 17.6	6–13, 10.0	85	Numerous
	Bastrichs and Mastigophores	13–18, 16.1	3–6, 3.4	18	Common	13–21, 16.5	2–8, 4.5	52	Numerous	12–21, 16.9	2–5, 3.4	37	Occasional
													
Mesenteries	Spirocysts	13–21, 17.4	2–6, 3.4	64	Numerous	13–25, 17.9	2–6, 3.3	60	Numerous	3–26, 18.0	2–5, 3.1	61	Numerous
	Holotrichs (L)	23–38, 28.2	7–14, 11.5	27	Occasional	20–34, 24.3	8–15, 10.8	31	Occasional	20–36, 27.6	10–15, 11.8	52	Numerous
	Holotrichs (M)	–	–	–		10–19, 16.5	4–15, 9.4	86	Numerous	12–19, 16.2	6–13, 8.3	61	Numerous
	Bastrichs and Mastigophores	13–18, 16.0	3–5, 3.8	16	Common	13–22, 16.9	3–9, 4.6	71	Numerous	10–18, 14.6	2–5, 2.7	21	Occasional

########### Habitat and distribution.

*Antipathozoanthus obscurus* sp. n. is found in low-light environments such as within crevasses of reef slopes and reef floors, and coral reef caves. Specimens were found from 3 to 15 m. This species has been found from the Red Sea and Okinawa.

########### Differential diagnosis.

*Antipathozoanthus obscurus* sp. n. is easily distinguished from all other *Antipathozoanthus* species, including the two other new species in this study, which all have associations with antipatharians. *A. obscurus* sp. n. is not associated with antipatharians and instead is found on coral reef carbonate substrate within caves or cracks. Additionally, the cnidome of *A. obscurus* sp. n. is different from all other known *Antipathozoanthus* species, including the other new species in this study, as there are no medium holotrichs in any tissue of *A. obscurus* sp. n., and instead only large holotrichs are found in all tissues.

Although *A. obscurus* sp. n. is not associated with antipatharians, phylogenetic data indicate that *A. obscurus* sp. n. is very closely related to other *Antipathozoanthus* species associated with antipatharians, with identical COI and 16S-rDNA sequences to those of *A. macaronesicus* (EU591618).

########### Remarks.

The samples of *Antipathozoanthus obscurus* sp. n. in the present study contain two morphotypes; one with bright brown tentacles that are longer than the oral disk (MISE-TF54); and the other morphotype with orange tentacles that are only as long as the oral disk (MISE-BISE1, MISE-BISE3, MISE-JDR190, MISE-JDR191, MISE-JDR192, MISE-JDR279, MISE-KU1, MISE-TF78, MISE-TF148). However, the sequences of all specimens formed a monophyletic clade and therefore we have described *A. obscurus* sp. n. in this study as containing two morphotypes. Genetic variation in all three genetic markers in the samples of *A. obscurus* sp. n. was observed, and the possibility remains that *A. obscurus* sp. n. may contain cryptic species. Thus, we have excluded specimen MISE-BISE3 from the type series, although it was tentatively identified as *A. obscurus* sp. n. Further specimens and fine-scale genetic analyses are required to better understand if there is any cryptic diversity within this species.

########### Etymology.

*Antipathozoanthus obscurus* sp. n. is named from the Latin “obscura" meaning “dark", as this species can be found in dark environments.

########### Common name.

Tsuno-nashi-sunaginchaku (new Japanese name).

########## Antipathozoanthus remengesaui sp. n.

Taxon classificationAnimaliaZoanthariaParazoanthidae

http://zoobank.org/758A60AA-6D66-441A-8D54-2181F5ACF48D

Fig. 2c

Antipathozoanthus sp. sensu Reimer et al. 2014a, 2, fig. 1d
Antipathozoanthus sp. 1 sensu Reimer and Fujii 2017, 304, fig. 14.4c.

########### Material examined.

*Holotype*: NSMT-Co1603 (MISE-PALAU2), colony of approximately 70 polyps connected by poorly developed white coenenchyme on genus *Antipathes* antipatharian (Hexacorallia: Antipatharia: Antipathidae). Preserved polyps approximately 1.5–3.0 mm in diameter, and approximately 1.5–2.0 mm in height from coenenchyme. Collected from Blue Hole, Palau (7°8'29.4"N, 134°13'23.3"E) at a depth of 23 m by JDR, 15 September 2014.

*Paratypes*: RMNH.Coel.42321 (MISE-MAL84), collected from Wall Street, Maldives (3°07'14.2"N, 72°58'46.5"E) at a depth of 9 m by JDR, 7 May 2014; RUMF-ZG-4397 (MISE-MAL85), collected from Wall Street, Maldives (3°07'14.2"N, 72°58'46.5"E) at a depth of 9 m by JDR, 7 May 2014; RUMF-ZG-4398 (MISE-JDR209), collected from Yanbu, Saudi Arabia, (24°26'N, 37°14'E) at a depth of 11 m by JDR, 4 October 2013; RUMF-ZG-4399 (MISE-TF102), collected from Okoga-shima Island, Kagoshima, Japan (31°33'58.75"N, 130°35'32.01"E) at a depth of 20 m by TF, 26 July 2011; RUMF-ZG-4400 (MISE-TF103), collected from Okoga-shima Island, Kagoshima, Japan (31°33'58.75"N, 130°35'32.01"E) at a depth of 40 m by TF, 26 July 2011.

*Other materials examined*: MISE-PALAU3, collected from Siaes Tunnel, Palau (7°18'54.8"N, 134°13'13.3"E) at a depth of 37 m by JDR, 15 September 2014; MISE-PALAU4, collected from Blue Hole, Palau (7°8'29.4"N, 134°13'23.3"E) at a depth of 28 m by JDR, 12 September 2014; MISE-KINKO2, collected from Hakamagoshi, Sakurajima, Kagoshima, Japan (31°35'23.5"N, 130.35.27.8"E) at a depth of 21 m by JDR, 20 September 2015; MISE-TF173, collected from Onna, Okinawa, Japan (26°26'20.9"N, 127°47'7.22"N) at depth of 15 m by TF, 27 June 2014; MISE-MAL46, collected from Coral Garden, Maldives (3°05'24.3"N, 72°58'04.5"E) at a depth of 24 m by JDR, 6 May 2014; MISE-MAL82, collected from Wall Street, Maldives (3°07'14.2"N, 72°58'46.5"E) at a depth of 9 m by JDR, 7 May 2014; MISE-MAL83, collected from Wall Street, Maldives (3°07'14.2"N, 72°58'46.5"E) at a depth of 9 m by JDR, 7 May 2014; MISE-MAL2502602, collected from Capital Reef, Maldives (3°02'55.8"N, 72°53'21.2"E) at a depth of 19 m by Marco Oliverio, 16 May 2014; MISE-MAL145, collected from Wall Street, Maldives (3°07'14.2"N, 72°58'46.5"E) at a depth of 12 m by JDR, 10 May 2014; MISE-MAL147, collected from Wall Street, Maldives (3°07'14.2"N, 72°58'46.5"E) at a depth of 10 m by JDR, 10 May 2014; MISE-MAL261, collected from Wall Street, Maldives (3°07'14.2"N, 72°58'46.5"E) at a depth of 9 m by JDR, 17 May 2014; MISE-HK70, collected from Siaes Tunnel, Palau (7°18'54.8"N, 134°13'13.3"E) by Hiroki Kise (HK), 12 September 2014, depth not available; MISE-HK90, collected from Blue Hole, Palau (7°8'29.4"N, 134°13'23.3"E) at a depth of 22 m by HK, 15 September 2014; MISE-JDR211, collected from Yanbu, Saudi Arabia, (24°26'N, 37°14'E) at a depth of 12 m by JDR, 4 October 2013; MISE-JDR214, collected from Yanbu, Saudi Arabia, (24°26'N, 37°14'E) at a depth of 12 m by JDR, 4 October 2013.

########### Diagnosis.

*External morphology*: Polyps *in situ* are approximately 4–8 mm in diameter, and approximately 3–8 mm in height *in situ* when oral disks expanded (Fig. 2). Colonial zoantharian, white or off-white polyps that may be solitary or connected by a white and poorly developed coenenchyme on *Antipathes* substrate. *Antipathozoanthus remengesaui* sp. n. has approximately 40–42 tentacles that are pinkish or/and translucent. Tentacles are usually as long as open oral disk diameter. Oral disk is pink or bright brown in color, and the capitulum is also pinkish or bright brown in color when polyps are closed. Polyps encrusted with visible sand particles (1–3 mm) in their coenenchyme and ectodermal tissue. Colonies attached on axis from proximal extremity to base of *Antipathes*.

**Figure 4.**
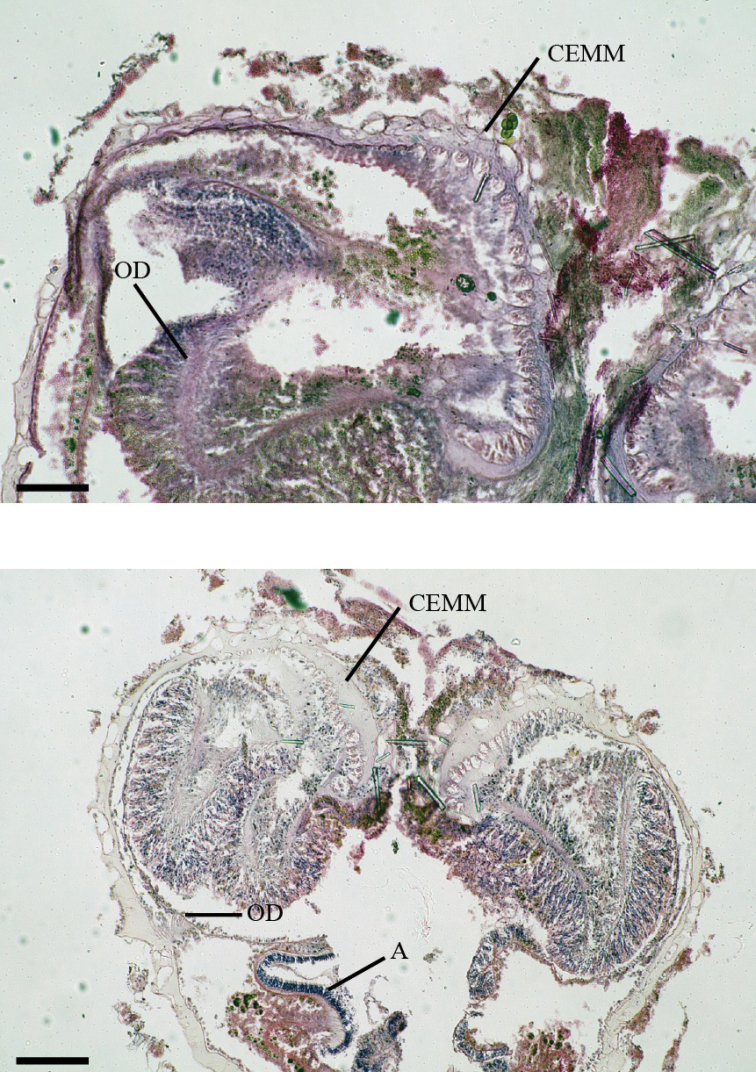
Images of histological section of *Antipathozoanthus* species. **a** longitudinal section of *A. remengesaui* sp. n. **b** longitudinal section of *A. cavernus* sp. n.. Abbreviations: (CEMM) cteniform endodermal marginal muscle, (OD) oral disk, (A) actinopharynx. Scale bars: **a** 200 µm, **b** 50 µm.

*Internal morphology*: Cteniform endodermal marginal muscle sensu Swain et al. 2015 (Fig. 4). Azooxanthellate. The large scattered lacunae in ectoderm and mesogleal are present due to their encrustations.

*Cnidae*: Holotrichs (large and medium), basitrichs and microbasic p-mastigophores (usually difficult to distinguish), spirocysts (Fig. 3; Table 2).

########### Habitat and distribution.

*Antipathozoanthus remengesaui* sp. n. has been found on the sides and/or floors of cave entrance, and always on *Antipathes*. Specimens were collected from depths of 9 to 40 m. This species is known from Palau, Kagoshima in Japan, the Maldives, and the Red Sea.

########### Differential diagnosis.

In the Pacific, *Antipathozoanthus remengesaui* sp. n. can be distinguished from *A. hickmani* by the development of the coenenchyme and in part by polyp size; the larger polyps (4–12 mm in diameter and 4–15 mm in height) of *A. hickmani* are connected by a well-developed coenenchyme on *Antipathes galapagensis*, while the slightly smaller polyps (4–8 mm in diameter and 3–8 mm in height *in situ*) of *A. remengesaui* sp. n. are either connected by a poorly developed coenenchyme or may even be solitary on *Antipathes*. Additionally, the cnidomes of these species are different; *A. hickmani* does not have spirocysts in the column, while *A. remengesaui* sp. n. has spirocysts in the column.

########### Remarks.

The *Antipathozoanthus remengesaui* sp. n. specimens found in Kagoshima, Japan have different morphological features compared to the specimens found in all other regions. Specimens collected from Kagoshima have relatively large polyps (6–8 mm in diameter, and approximately 5–8 mm in height *in situ*) compared to specimens from other regions. The coloration of oral disks is also different between Kagoshima and other regions; *A. remengesaui* sp. n. from Kagoshima has a bright brown oral disk, while those from other regions have pink oral disks. However, sequences of these specimens collected from all regions formed a monophyletic clade for all genetic markers including ITS-rDNA. In terms of substrate organisms, *A. remengesaui* sp. n. collected from all regions in this study was associated with black corals of the genus *Antipathes*. Here, we have described this group as a single species, *A. remengesaui* sp. n., based on phylogeny and substrate specificity, although we have excluded some specimens for which we could not amplify ITS-rDNA successfully from the type series.

########### Etymology.

*Antipathozoanthus remengesaui* sp. n. is named after Tommy Esang Remengesau, Jr., the current president of the Republic of Palau, who has greatly contributed to marine research and conservation in Palau.

Common name. Momoiro-mame-tsuno-sunaginchaku (new Japanese name).

########## Antipathozoanthus cavernus sp. n.

Taxon classificationAnimaliaZoanthariaParazoanthidae

http://zoobank.org/CC4A5D45-FC91-4E8F-B496-184DDA7C1AC1

Fig. 2d

########### Material examined.

*Holotype*: NSMT-Co1604 (MISE-KINKO1), colony of approximately 125 polyps connected by a highly developed coenenchyme on genus *Myripathes* (Antipatharia: Myriopathidae). Preserved polyps approximately 2.0–5.0 mm in diameter, and approximately 2.0–5.0 mm in height from coenenchyme. Collected from Sakurajima, Kagoshima, Japan (31°35'23.5"N, 130.35.27.8"E) at a depth of 21 m by JDR, 20 September 2015.

*Paratypes*: RUMF-ZG-4401 (MISE-MAL2592601), collected from Capital Reef, Maldives (3°02'55.8"N, 72°53'21.2"E) at a depth of 19 m by Marco Oliverio, 16 May 2014; RMNH.Coel.42322 (MISE-PALAU5), collected from Siaes Tunnel, Palau (7°18'54.8"N, 134°13'13.3"E) at a depth of 39 m by JDR, 15 September 2014.

########### Diagnosis.

*External morphology*: Polyps *in situ* are approximately 4–15 mm in diameter when oral disk is expanded, and approximately 3–10 mm in height (Fig. 2). Colonial zoantharian with polyps connected by highly developed coenenchyme on *Myripathes*. *Antipathozoanthus cavernus* sp. n. has approximately 32–40 translucent tentacles of approximately 1 to 5 mm in length. Tentacle lengths are either as long as or slightly shorter than expanded oral disk diameter. Polyps have orange oral disk with orange or light orange ring around oral disk. When polyps are closed, capitular ridges are present and observed clearly, numbering approximately 16–20. The capitulum is orange or light orange in color. Polyps encrusted with visible sand particles (1–8 mm) in their coenenchyme and ectodermal tissue. Polyps usually much more encrusted than coenenchyme. Colonies attached on axis from proximal extremity to base of *Myripathes*.

*Internal morphology*: Cteniform endodermal arrangement marginal muscle sensu Swain et al. (2015) in longitudinal section (Fig. 4). Azooxanthellate. Large scattered lacunae in ectoderm and mesogleal are present due to their encrustations.

*Cnidae*: Holotrichs (large and medium), basitrichs and microbasic p-mastigophores (usually difficult to distinguish from each other), spirocysts (Fig. 3; Table 2).

########### Habitat and distribution.

*Antipathozoanthus cavernus* sp. n. is found on the sides and/or floor of cave entrances, and on steep slopes, and always on *Myripathes*. Specimens were collected from depths of 19 to 39 m.

########### Differential diagnosis.

*Antipathozoanthus cavernus* sp. n. occurs in similar environments as *A. remengesaui* sp. n., but these species can be distinguished by their coenenchyme development and by the generic identity of the antipatharian host. *A. remengesaui* sp. n. is associated with genus *Antipathes* (family Antipathidae) covered by a poorly developed coenenchyme, while *A. cavernus* sp. n. is associated with genus *Myripathes* (family ) covered by a highly developed coenenchyme. *A. cavernus* sp. n. can be distinguished from *A. hickmani* by a different coloration and by its antipatharian association; *A. cavernus* sp. n. does not have red or cream colored polyps as seen in *A. hickmani*. Additionally, *A. hickmani* is associated with *Antipathes galapagensis*, while *A. cavernus* sp. n. is associated with genus *Myripathes*. *A. macaronesicus* is easily distinguishable from *A. cavernus* sp. n. by their polyp coloration (orange and light orange versus pinkish and yellowish, and their antipatharian host (genus *Antipathes* versus genus *Myripathes*). Finally, all species above have unique ITS-rDNA sequences.

########### Etymology.

*Antipathozoanthus cavernus* sp. n. is named from the Latin “caverna" meaning “cave", as this species is found in caves.

########### Common name.

Hana-tsuno-sunaginchaku (new Japanese name).

### Phylogenetic analyses

**Figure 5.**
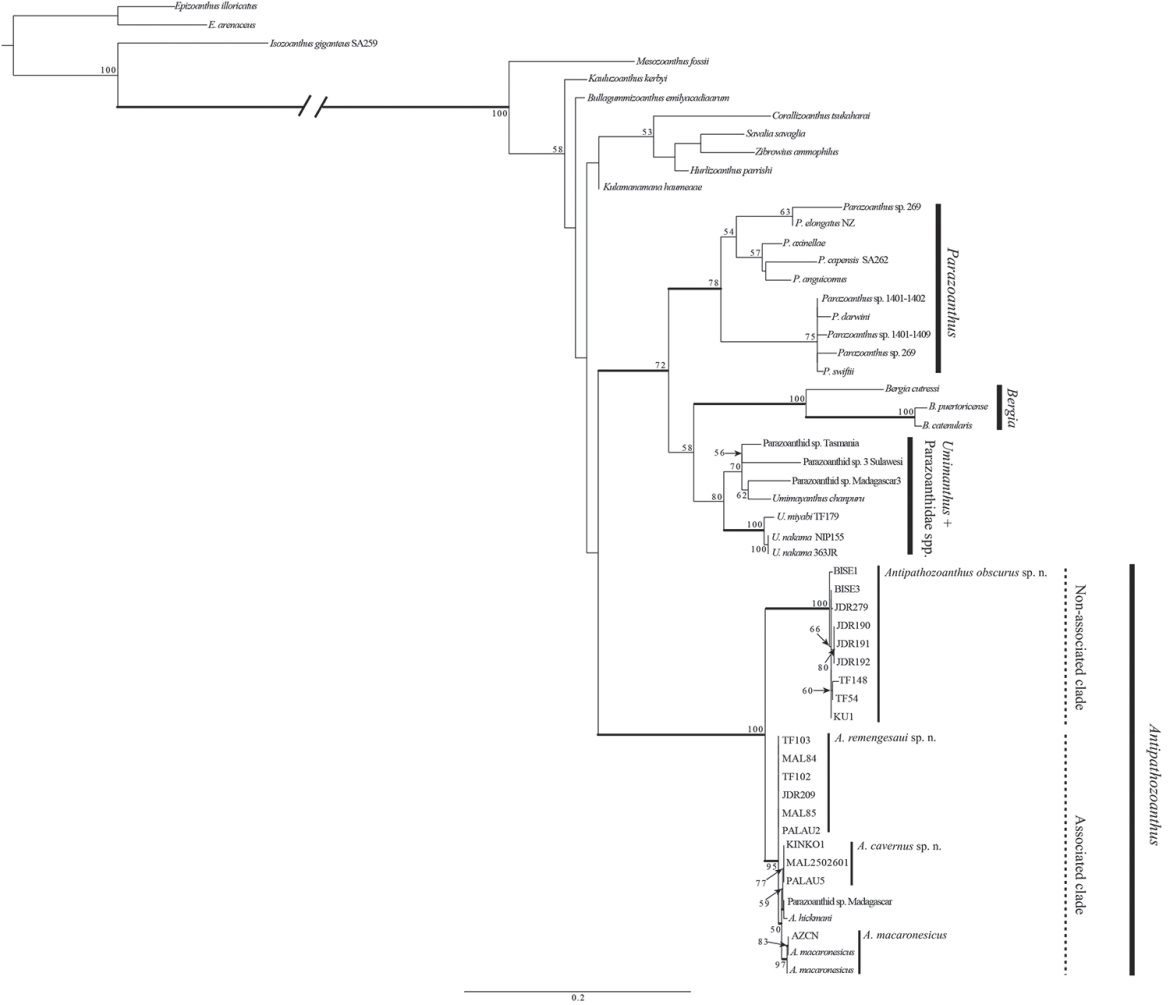
Maximum likelihood (ML) tree based on concatenated alignments of 16S-rDNA, COI and ITS-rDNA. Numbers on nodes represent ML bootstrap values (> 50% are shown). Bold branches indicate high supports of Bayesian posterior probabilities (> 0.95).

**Concatenated alignment.** All *Antipathozoanthus* species together formed a large monophyletic clade within the Parazoanthidae with complete support (ML = 100%, BI = 1) in the concatenated (COI+16S-rDNA+ITS-rDNA) alignment phylogeny (Fig. 5). Within the *Antipathozoanthus* clade, the various *Antipathozoanthus* species were divided into two subclades, an ‘associated’ subclade consisting of species associated with antipatharians, and a ‘non-associated’ subclade consisting only of *A. obscurus* sp. n. found directly on non-biotic substrates. The associated subclade consisted of *A. macaronesicus*, *A. hickmani*, *A. remengesaui* sp. n. and *A. cavernus* sp. n. and had very strong support (ML = 95%, BI = 0.99), while the non-associated subclade of *A. obscurus* sp. n. had complete support (ML = 100%, BI = 1). Within the associated clade, *A. hickmani* and *A. cavernus* sp. n. were sister to each other (ML = 59%, BI = 0.96). *A. remengesaui* sp. n. was basal to a poorly nodal supported clade (ML ≤ 50%, BI ≤0.95) containing other associated *Antipathozoanthus* spp. (*A. macaronesicus*, *A. hickmani* and *A. cavernus* sp. n.). *A. macaronesicus* formed a subclade with very strong support (ML = 97%, BI = 1).

**COI**. All *Antipathozoanthus* species formed a large monophyletic clade within the with a very strong support (NJ = 99%, ML = 99%, BI = 1) in the COI phylogeny (Suppl. material 2). Within the clade, *Antipathozoanthus* species were divided into two subclades (associated subclade + non-associated subclade). The topology within the large monophyletic associated subclade was very similar to that as seen in the 16Sr-DNA phylogeny. Both the associated subclade (NJ =77%, ML = 66%) and the non-associated subclade had moderate support (NJ = 86%, ML = 85%, BI = 0.99). Sequences of *Antipathozoanthus* species within each of the subclades showed no differences in sequences. The difference in sequences between the associated subclade and the non-associated subclade was 3 bp (0.69%).

**16S-rDNA.** All *Antipathozoanthus* species formed a large monophyletic clade within the Parazoanthidae with generally high support (NJ = 99%; ML = 85%; BI = 1) in the 16Sr-DNA phylogeny (Suppl. material 3). Within this large clade, *Antipathozoanthus* species were divided into two subclades; an associated subclade (*A. macaronesicus*, *A. hickmani*, *A. remengesaui* sp. n., and *A. cavernus* sp. n.); and the other subclade not associated with antipatharians (*A. obscurus*. sp. n.; ‘non-associated subclade’). The associated subclade formed only in NJ phylogenetic tree with moderate support in the 16S-rDNA tree (NJ = 78%), while the non-associated subclade had strong support in each phylogeny (NJ = 97%; ML = 95%; BI = 0.96). Sequences of *Antipathozoanthus* species within the associated subclade were identical with the exception of *A. hickmani* (EU333757), which differed by one base substitution, while within the non-associated subclade there were a few small sequence differences (maximum difference 3 bp). Differences of sequences between the associated and the non-associated subclades were 4–6 bp (0.67 to 1.01%).

**ITS-rDNA.** All *Antipathozoanthus* species formed a large monophyletic clade within the Parazoanthidae with complete support (NJ = 100%, ML = 100%, BI = 1) in the ITS-rDNA phylogeny (Suppl. material 4). Within the *Antipathozoanthus* clade there were again two subclades, corresponding to the associated subclade and the non-associated subclade, as seen in both the mitochondrial COI and 16S-rDNA phylogenies. The associated subclade had moderate support (NJ = 100%, ML = 69%, BI = 0.96), while the non-associated subclade had very strong support (NJ = 96%, ML = 100%, BI = 1). Within the associated subclade, all four species had different sequences; *A. macaronesicus* formed a monophyletic grouping with very strong support (NJ = 99%, ML = 90%, BI = 0.99), while *A. remengesaui* sp. n., *A. cavernus* sp. n., and *A. hickmani* each formed monophyletic groupings with moderate support (NJ = 95%, ML = 69%, BI = 0.62; NJ = 86%, ML = 62%, BI = 0.96; NJ = 79%; ML = 84%, BI = 0.96, respectively). *A. obscurus* sp. n. formed a monophyletic clade with very strong support (NJ = 96%; ML = 100%; BI = 1).

## Discussion

**Distribution of *Antipathozoanthus* species in the Indo-Pacific Ocean.** *Antipathozoanthus hickmani* is found in only the Galapagos with A. cf. hickmani reported from the coast of Ecuador (Bo et al. 2012), suggesting an East Pacific distribution. On the other hand, *A. remengesaui* sp. n. was found in the Red Sea, the Maldives, Palau, and mainland Japan and Okinawa, Japan, while *A. cavernus* sp. n. was found in the Maldives, Palau, and mainland Japan, and *A. obscurus* sp. n. was found in the Red Sea and Okinawa, Japan. Additionally, unidentified *Antipathozoanthus* species have been previously reported from the central Indo-Pacific Ocean (Reimer et al. 2014a), the South China Sea (Reimer et al. 2017), and mainland Japan (Reimer et al. 2013). These results indicate that the three new *Antipathozoanthus* species described herein are likely widely distributed across the Indo-Pacific Ocean, and also that *Antipathozoanthus* species diversity is higher than has been previously known.

**Evolution of macrocnemic zoantharians in caves**. *Antipathozoanthus obscurus* sp. n. without host was found in similar environments as the ‘associated’ *Antipathozoanthus* species, but this species does not associate with antipatharians and is instead directly attached to coral reef carbonate. Ocaña and Brito (2003) explained the relationship between *Antipathozoanthus* and antipatharians as a case of facultative parasitism, although this association still requires further research. It has been revealed that some macrocnemic species gain an advantage in plankton feeding by utilizing substrate organisms that filter feed in environments where plankton organisms are scarce (e.g., *Hydrozoanthus* species on oligotrophic coral reefs; Di Camillo et al. 2010), and this could be one reason that most *Antipathozoanthus* spp. utilize antipatharians as substrate. However, moderate currents conducive to plankton feeding may occur in coral reef caves by inflow of tidal currents or terrestrial runoff (Iliffe and Kornicker 2009), and it may be unnecessary to have an association with antipatharians for obtaining sufficient plankton in such environments. Additionally, in marine caves, there are fewer predators of zoantharians, such as fishes (e.g., Bussotti et al. 2002), and perhaps fewer competitors for substrate space. Such environments may promote the speciation of ‘non-associated’ zoantharian species as seen here with *A. obscurus* sp. n.

All new species in the present study are azooxanthellate, and this trait is common within macrocnemic zoantharians to the exception of some species such as *Bergia cutressi* (West, 1979) and *Nanozoanthus harenaceus* Fujii & Reimer, 2013. Irei et al. (2015) suggested that cave-dwelling *Palythoa* species within Brachycnemina lost their zooxanthellae to adapt to environments in caves and cracks. On the other hand, macrocnemic cave-dwelling species may originally have lacked zooxanthellae rather than undergone a loss of zooxanthellae. However, more investigations are needed to evaluate the species diversity of zoantharians in caves to more comprehensively understand the evolution of these zoantharian species.

**Substrate specificity within *Antipathozoanthus*.** Within the family Parazoanthidae, different generic lineages likely have long evolutionary histories associated with their substrate organisms, based on the fact that many parazoanthid genera form monophyletic clades in accord to their substrates (Sinniger et al. 2005, 2010, 2013; Montenegro et al. 2015). In this study, we found two different subclades within *Antipathozoanthus* (Fig. 5, Suppl. Materials 2–4) that corresponded to substrate differences. The genetic distances between the associated subclade (*A. hickmani*, *A. macaronesicus*, *A. remengesaui* sp. n., *A. cavernus* sp. n.) and the non-associated subclade of *A. obscurus* sp. n. were 0.60% (COI) to 1.01% (16S-rDNA). Additionally, we observed characteristic insertions and deletions in the 16S-rDNA between the associated and non-associated subclades. Although the two clades formed in accordance to their substrate (antipatharians, coral reef carbonate), we consider the genetic distances between the two clades as intra-generic based on previous comparisons of genetic distances (Sinniger et al. 2010). While many taxonomic and molecular studies focusing on the family Parazoanthidae have been conducted using various genetic markers (e.g., Sinniger et al. 2005, 2010; Reimer et al. 2008; Montenegro et al. 2015), little research has been conducted focusing on the phylogenetic relations within different parazoanthid genera, except for studies examining *Bergia, Parazoanthus*, and *Umimayanthus*, which are all associated with sponges (Sinniger et al. 2005; Montenegro et al. 2015, 2016; Carreiro-Silva et al. 2017). There is a need for more phylogenetic studies focusing on increasing the numbers of species examined within each of the genera of Parazoanthidae in order to better understand the evolutionary history of substrate specificity and other traits within the family Parazoanthidae (Swain et al. 2015, 2016).

## Supplementary Material

XML Treatment for Antipathozoanthus

XML Treatment for Antipathozoanthus obscurus

XML Treatment for Antipathozoanthus remengesaui

XML Treatment for Antipathozoanthus cavernus

## Supplementary materials

Supplementary material 1 List of GenBank accesion number Data type: GenBank accession numbers Explanation note: GenBank accession numbers, names and details of the sequences used in phylogenetic analyses of COI, 16S-rDNA and ITS-rDNA in this study. Sequences that were concatenated are indicated by bold text. This is an open access article distributed under the terms of the Creative Commons Attribution License (CC BY 4.0), which permits unrestricted use, distribution, and reproduction in any medium, provided the original author and source are credited.Hiroki Kise, Takuma Fujii, Giovanni Diego Masucci, Piera Biondi, James Davis Reimer

Supplementary material 2 Phylogenetic tree of COI Data type: phylogenetic tree Explanation note: Maximum likelihood (ML) tree based on cytochrome oxidase subunit I sequences. Numbers on nodes represent ML and neighbor-joining (NJ) bootstrap values (> 50% are shown). Bold branches indicate high supports of Bayesian posterior probabilities (> 0.95). This is an open access article distributed under the terms of the Creative Commons Attribution License (CC BY 4.0), which permits unrestricted use, distribution, and reproduction in any medium, provided the original author and source are credited.Hiroki Kise, Takuma Fujii, Giovanni Diego Masucci, Piera Biondi, James Davis Reimer

Supplementary material 3 Phylogenetic tree of 16S-rDNA Data type: phylogenetic tree Explanation note: Maximum likelihood (ML) tree based on mitochondrial 16S ribosomal DNA sequences. Numbers on nodes represent ML and neighbor-joining (NJ) bootstrap values (> 50% are shown). Bold branches indicate high supports of Bayesian posterior probabilities (> 0.95). This is an open access article distributed under the terms of the Creative Commons Attribution License (CC BY 4.0), which permits unrestricted use, distribution, and reproduction in any medium, provided the original author and source are credited.Hiroki Kise, Takuma Fujii, Giovanni Diego Masucci, Piera Biondi, James Davis Reimer

Supplementary material 4 Phylogenetic tree of ITS-rDNA Data type: phylogenetic tree Explanation note: Maximum likelihood (ML) tree based on internal transcribe spacer region of ribosomal DNA sequences. Numbers on nodes represent ML and neighbor-joining (NJ) bootstrap values (> 50% are shown). Bold branches indicate high supports of Bayesian posterior probabilities (> 0.95). This is an open access article distributed under the terms of the Creative Commons Attribution License (CC BY 4.0), which permits unrestricted use, distribution, and reproduction in any medium, provided the original author and source are credited.Hiroki Kise, Takuma Fujii, Giovanni Diego Masucci, Piera Biondi, James Davis Reimer
