# Supplementary figures and images for: Three new species and the molecular phylogeny of Antipathozoanthus from the Indo-Pacific Ocean (Anthozoa, Hexacorallia, Zoantharia)
Source: Zookeys. 2017 Dec 29;(725):97–122. doi: 10.3897/zookeys.725.21006 (PMC5769718; doi:10.3897/zookeys.725.21006)

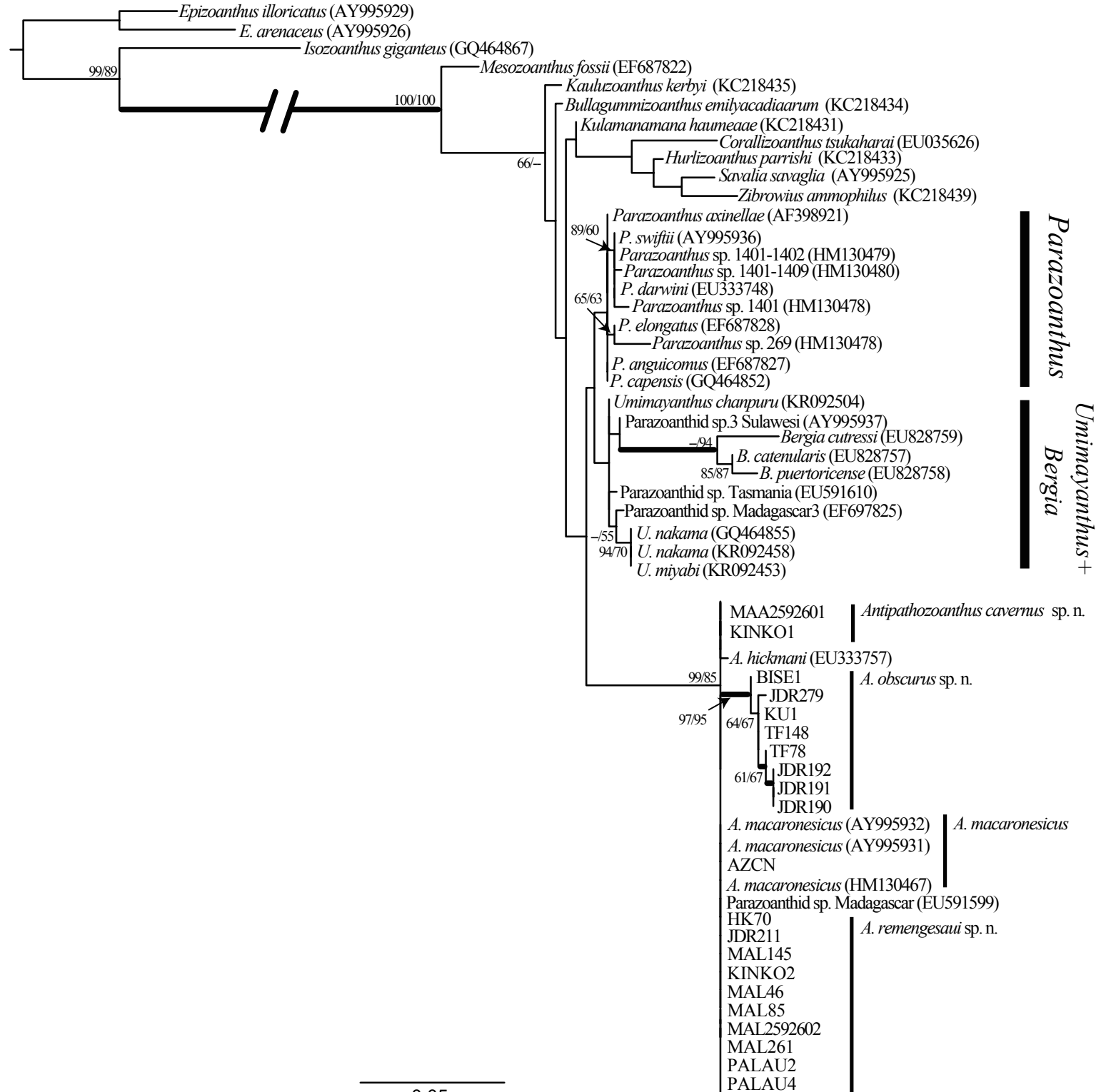

Antipathozoanthus

Supplement: Supplementary material 2 — Phylogenetic tree of COI [file zookeys-725-097-s002.pdf]

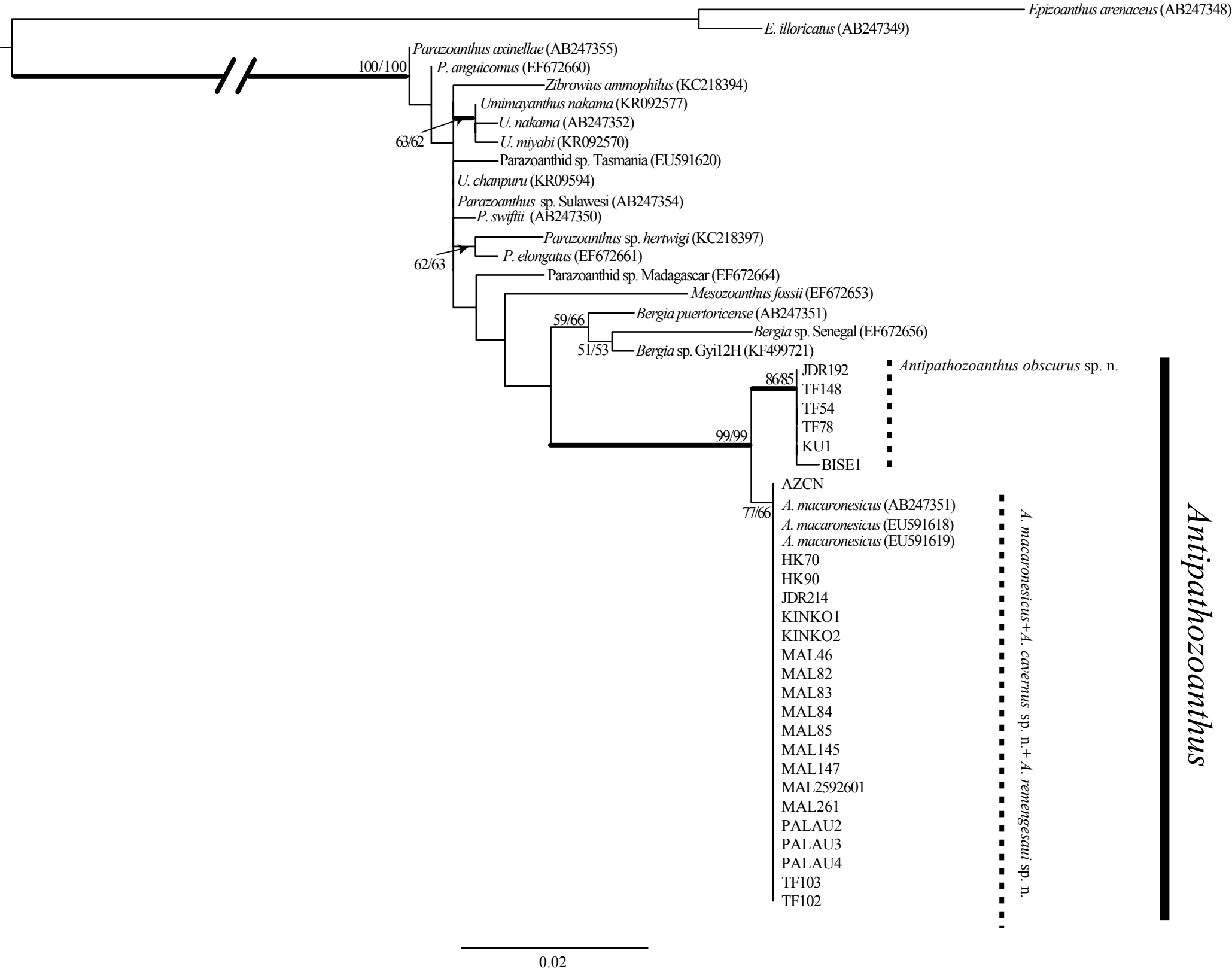

Supplement: Supplementary material 3 — Phylogenetic tree of 16S-rDNA [file zookeys-725-097-s003.pdf]

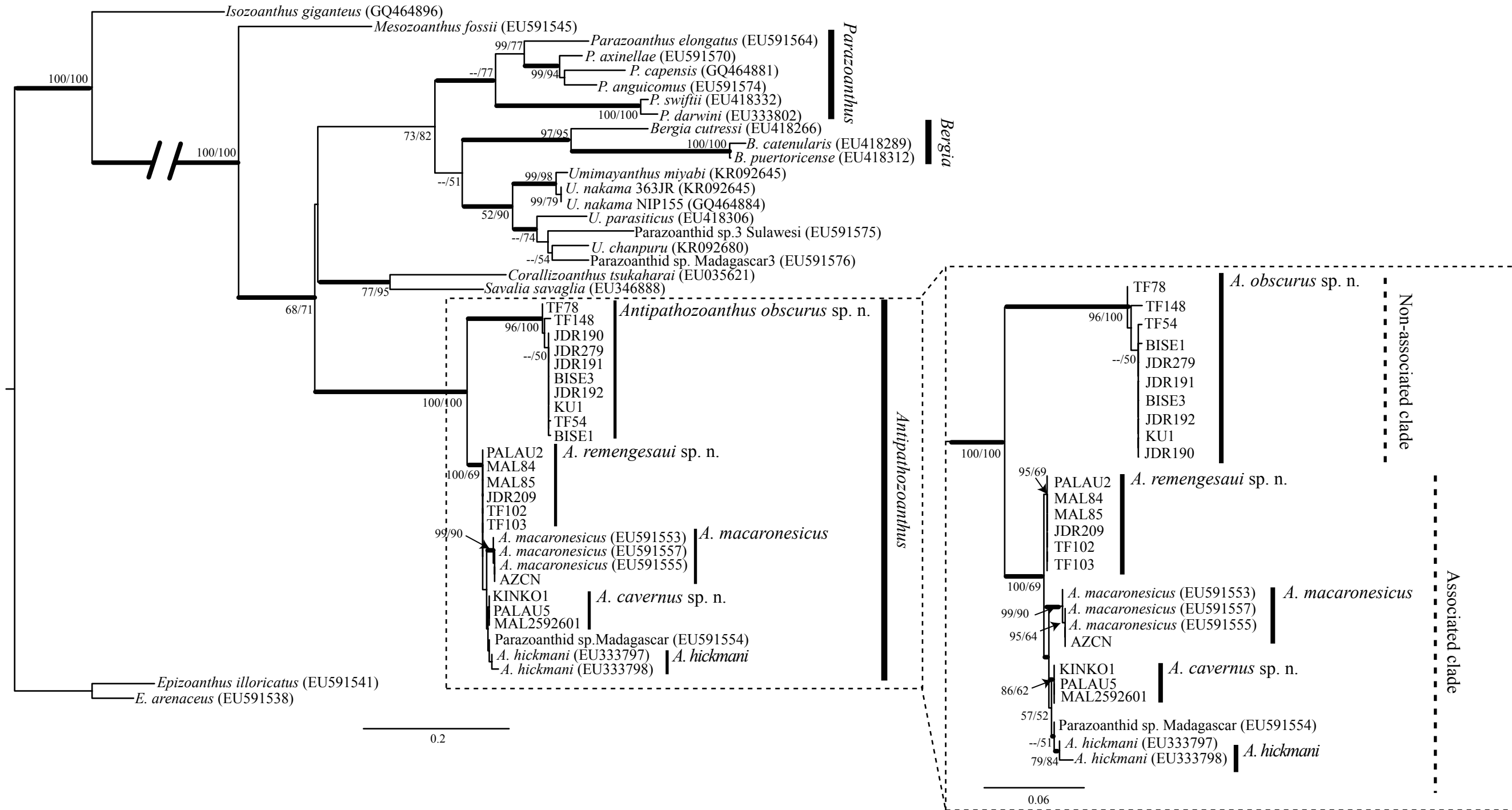

Supplement: Supplementary material 4 — Phylogenetic tree of ITS-rDNA [file zookeys-725-097-s004.pdf]
